# Increasing Phylogenetic Clustering of Arbuscular Mycorrhizal Fungal Communities in Roots Explains Enhanced Plant Growth and Phosphorus Uptake

**DOI:** 10.1007/s00248-024-02457-1

**Published:** 2024-11-14

**Authors:** Adam Frew, Carlos A. Aguilar-Trigueros

**Affiliations:** 1 Hawkesbury Institute for the Environment, Western Sydney University, Hawkesbury Campus, Locked Bag 1797, Penrith, 2751 NSW Australia; 2 Centre for Crop Health, University of Southern Queensland, Toowoomba, 4350 QLD Australia; 3 Department of Biological and Environmental Sciences, University of Jyväskylä, P.O. Box 35, Jyväskylän yliopisto, FI-40014 Finland

**Keywords:** Arbuscular mycorrhiza, Community assembly, Phylogenetic diversity, *Sorghum bicolor*

## Abstract

Temporal variation during the assembly of arbuscular mycorrhizal (AM) fungal communities within plant roots have been posited as critical drivers of the plant-fungal symbiotic outcomes. However, functional implications of these dynamics for the host plant remain poorly understood. We conducted a controlled pot experiment with *Sorghum bicolor* to investigate how temporal shifts in AM fungal community composition and phylogenetic diversity influence plant growth and phosphorus responses to the symbiosis. We characterised the root-colonising AM fungal communities across three time points and explored their community assembly processes by analysing their phylogenetic diversity and employing joint species distribution modelling with the Hierarchical Modelling of Species Communities (HMSC) framework. We found strong AM fungal turnover through time with a high phylogenetic signal, indicating recruitment of phylogenetically clustered AM fungal species in the host. This temporal phylogenetic clustering of communities coincided with marked increases in plant biomass and phosphorus responses to the AM fungal symbiosis, suggesting that host selection for specific fungi may be a key determinant of these benefits.

**Supplementary Information:**

The online version contains supplementary material available at 10.1007/s00248-024-02457-1.

Most terrestrial plants engage in symbiotic associations with arbuscular mycorrhizal (AM) fungi [1]. In this symbiosis, the fungi colonise plant roots and the surrounding soil, facilitating the plant’s access to essential nutrients such as phosphorus, while acquiring carbon from the plant [2]. The composition of AM fungal communities exerts a considerable influence on the symbiotic effects experienced by the plant hosts [3–5]. Consequently, understanding the determinants of AM fungal community composition has long been a focus of ecological research [6–8]. However, achieving this is complicated by the global distribution of AM fungi, whose taxa are found across multiple hosts and a wide array of environmental conditions [9].

Despite their widespread distribution, evidence suggests that environmental filtering plays a critical role in the assembly of AM fungal communities [9]. Local-scale studies often reveal that these assemblages are composed of closely related individuals, suggesting that phylogenetic clustering occurs as communities change to suit specific habitats [10]. In the context of root-colonising communities, the root system of the host plant serves as the local habitat for the fungi. These root-colonising communities are frequently observed to be phylogenetically clustered [7], likely as a result of both abiotic filtering and mutualistic partner selection [11–13]. With evidence that plant hosts can show preference toward more beneficial fungal taxa [14–16], it may be presumed that such partner selection would lead to AM fungal assemblages that confer greater functional benefits to the host plant compared to communities assembled in a purely stochastic manner. Based on this assumption, it is reasonable to expect that newly assembled AM fungal communities, such as in roots of seedlings, would become increasingly phylogenetically clustered over time as host-fungal compatibility is optimised to maximise symbiotic benefits.

The specific mechanisms through which host plants influence the assembly of AM fungal communities, particularly in terms of selecting taxa, remain poorly understood. Host affinity can be examined using different approaches such as the assessment of differential carbon allocation by hosts to more beneficial fungi [16], direct measurements of plant fitness in response to specific AM fungal taxa [17], or through comprehensive sampling of multiple host species within a given region [11]. While such studies can highlight that host preference could be a potential driver of community composition, a challenge remains in consistently identifying which fungal taxa provide the greatest benefits to their hosts. This challenge is compounded by the context-dependent nature of symbiotic outcomes, leading to significant variability in the functional roles of different AM fungal lineages [18, 19].

Assigning specific functional characteristics to different AM fungal taxa, particularly in terms of the potential ‘benefits’ they provide to hosts, has proven challenging in itself due to the highly context-dependent nature of these outcomes [5] and the difficulties of measuring traits on fungal individuals [20]. Despite decades of research, data associating particular AM fungal lineages with specific symbiotic effects on plant performance, remain limited [5, 21]. Notwithstanding this variability, it is generally accepted that AM fungi exhibit phylogenetic niche conservatism [22], that species retain ecological traits and niches over time. Consequently, closely related AM fungal taxa tend to exhibit similar characteristics, which could be anticipated to result in similar symbiotic effects on a given host [22].

Although AM fungal community composition significantly influences symbiotic outcomes for host plants [3, 5], and temporal dynamics have been observed in AM fungal spore populations and root colonisation [23, 24], comparatively few studies have directly investigated the temporal dynamics of AM fungal community composition within plant roots [10]. Those studies which have examined this, report significant changes in community composition over time [25–29] (but see [30]), often linked to host developmental stages or shifts in edaphic variables. The most detailed of these studies observed a shift from stochastic to deterministic assembly processes, with root communities exhibiting increased phylogenetic clustering through time [25]. This was attributed to the expanding available habitat—the growing root system—facilitating the immigration of fungal taxa with similar ecological niche requirements, resulting in communities composed of more closely related fungi. Despite being conducted in a relatively homogeneous agricultural context, this study would nevertheless have been influenced by soil heterogeneity and the natural spatial variation in the resident soil AM fungal communities, as well as potential dispersal effects. Consequently, additional data are required to enhance our understanding of the temporal dynamics of AM fungal communities in plant roots. It also remains unclear whether, or how, temporal shifts in the composition or phylogenetic structure of root-colonising communities have functional consequences in terms of their symbiotic effects on the host.

We conducted a glasshouse pot experiment with 60 individual plants of *Sorghum bicolour* L. Moench cv. ‘MR. Bazley’ with the objectives of (i) characterising temporal changes in AM fungal community composition and phylogenetic diversity within plant roots, and (ii) determining how these temporal changes relate to plant growth and phosphorus uptake (see Supplementary Information for methodological details). We hypothesised that (i) communities would become more phylogenetically clustered over time and (ii) this would correlate with an increase in the growth and phosphorus benefits provided by the symbiosis to the host.

Plants were cultivated in a fully homogenised and gamma-irradiated sand-soil mixture, which was either inoculated with a homogenised diverse community of AM fungi (AM fungi treatment) or with a sterilised inoculum of the same (No AM fungi treatment). The AM fungal community was sourced from a combination of field soils collected from various agricultural and non-agricultural sites previously known to support a high diversity of AM fungi (Fig. S5a). Plants were harvested at 4, 8, and 12 weeks (timepoints one, two, and three, respectively), where 20 replicate plants (10 with AM fungi and 10 without) were harvested. Total biomass was measured, foliar samples were collected for nutrient analysis, and root samples were subjected to Illumina amplicon sequencing using the nuclear small subunit (SSU) rRNA gene to identify AM fungal virtual taxa (VT) [31] and characterise community composition. Changes in AM fungal communities over time were analysed using a joint species distribution Bayesian framework, Hierarchical Modelling of Species Communities (HMSC; [32]). We employed this framework for the ability to model multiple species simultaneously while accounting for phylogenetic relationships, species interactions, and hierarchical data structures. Unlike classic regression methods that model species independently and may overlook inter-species interactions, or standard multivariate analyses that lack species-specific insights and explicit consideration of phylogeny, HMSC provides a comprehensive framework. This allows us to partition variance among fixed effects (e.g., timepoint, sequencing depth), random effects (e.g., individual plant variability), and phylogenetic contributions, offering deeper ecological insights into the factors shaping the community than traditional methods. We used this approach in combination with calculating beta diversity metrics alongside phylogenetic indices [33] measuring the extent of phylogenetic clustering or overdispersion. The AM fungal community dynamics were then assessed in relation to plant biomass and phosphorus concentrations to explore if and how temporal AM fungal assembly was associated with symbiotic outcomes for the host.

Our HMSC model demonstrated high discrimination ability (mean AUC = 0.88), reasonable accuracy (mean RMSE = 0.25), and modest explanatory power (mean Tjur’s R^2^ = 0.2) overall. The model evaluated the occurrence of AM fungal VT across the three timepoints and exhibited strong support for taxon-specific responses, particularly towards the third time point (Fig. 1a). Overall, the model attributed 45.3% of the explained variation in AM fungal occurrence to time (Fig. 1b), with 29.5% to the random effect of individual samples. Our model also attributed 25.2% to sequencing depth, indicating that the number of sequences generated per sample influenced the detected taxa. Including sequencing depth in our model allows us to account for this influence and improves our ability to isolate and interpret the true ecological patterns in the data. Our HMSC model had a notably high phylogenetic signal (ρ = 0.84 ± 0.0037; mean ± SE), indicating that the phylogenetic relatedness strongly predicts which AM fungal taxa are present at a given timepoint. This suggests that species traits conserved through evolutionary history play an important role in community assembly over time [32]. Indeed, of the 36 AM fungal taxa (VT) significantly associated with timepoint three, 35 belonged to the Glomeraceae family (Fig. 1a).

**Fig. 1:**
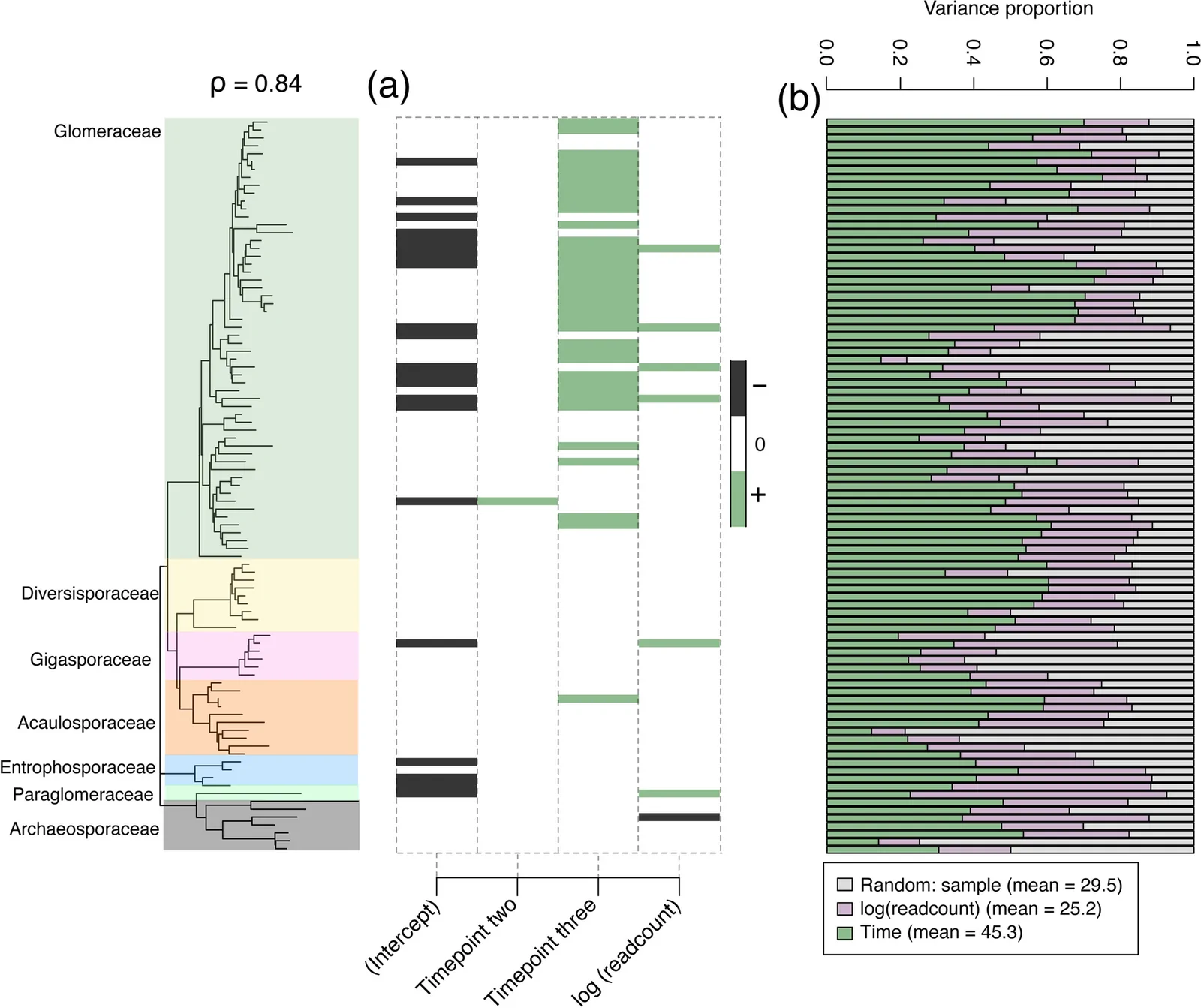
Hierarchical Modelling of Species Communities (HMSC) (**a**) beta coefficients indicating positive (green), negative (black), or no significant relationship (blank/white) of arbuscular mycorrhizal (AM) fungal virtual taxa (VT) responses with at least a posterior probability of 0.95 associated with timepoints two, three, and sampling depth (log readcount). The mean Rho (ρ) of the model, as a measure of phylogenetic signal in species’ responses, is shown. **b** The proportion of explained variation in AM fungal VT occurrence by time, sampling depth (log readcount), and the random effect of sample identity. Phylogenetic tree coloured by family is shown which includes the detected AM fungi across all samples, the AM fungal VT in (a) and (b) are sorted vertically according to their phylogenetic relatedness

Temporal shifts in community composition revealed high species turnover, with fungi from families such as Entrophosporaceae, Archaeosporaceae, and Diversisporaceae present at earlier timepoints but mostly absent by the third. Correspondingly, the variation in community composition among samples (the community dispersion) was lowest at the third timepoint (Fig. S2), reflecting increasing similarity among communities through time. The phylogenetic diversity also exhibited distinct trends, with standardised effect sizes of mean pairwise distances and mean nearest taxon distances significantly decreasing from the first to the third timepoint (Fig. 2a, b). These more negative values reflect a trend towards phylogenetic clustering suggesting that, as the AM fungal communities assembled over time, they became composed of more closely related taxa. This pattern is often thought to be indicative of community assembly processes driven by some form of environmental filtering [34, 35].

**Fig. 2:**
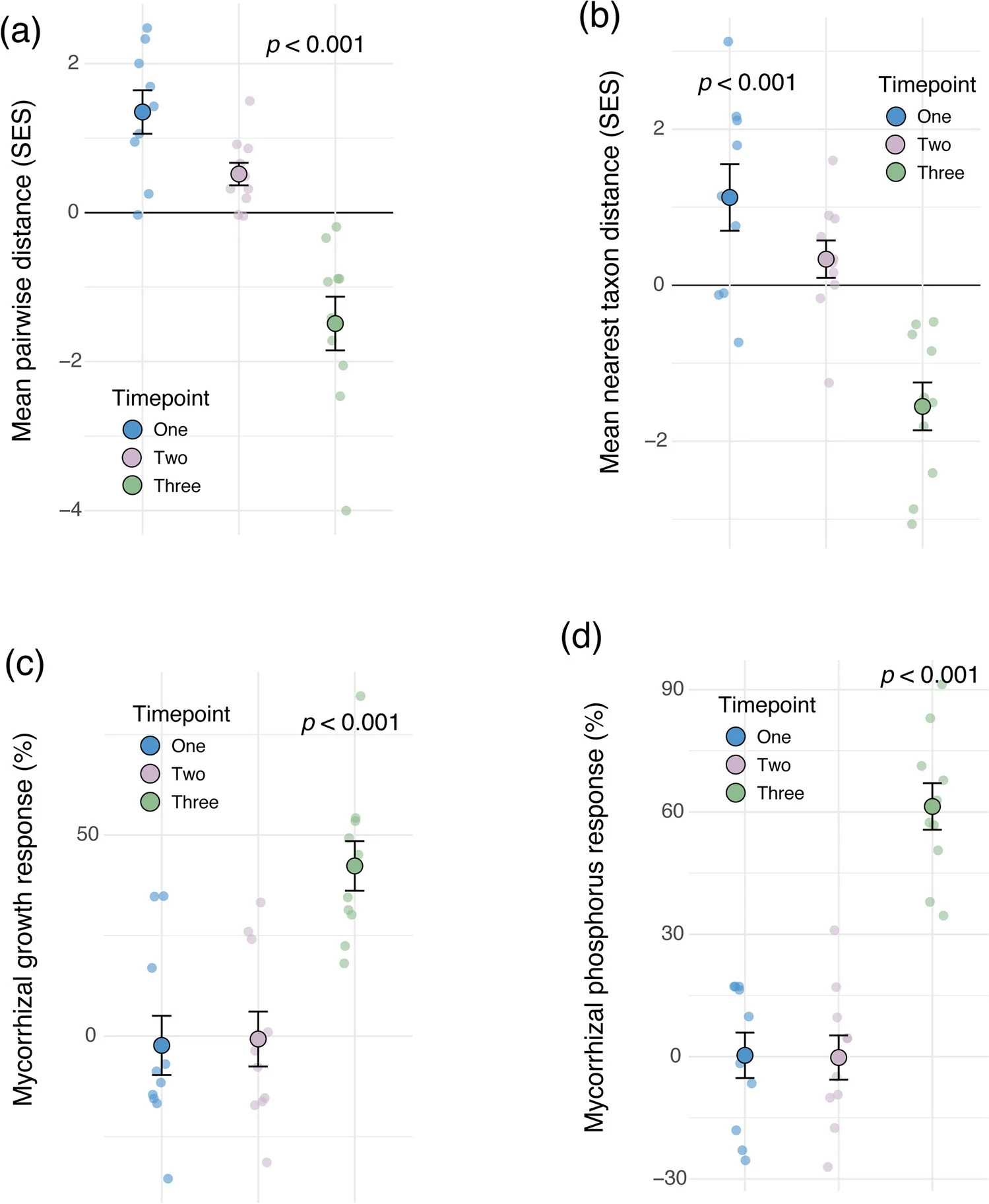
Phylogenetic diversity of root-colonising arbuscular mycorrhizal (AM) fungal communities as standardised effect sizes (SES) of (**a**) the mean pairwise distances and (**b**) mean nearest taxon distances at timepoints one, two, and three. The (**c**) mycorrhizal growth responses (%), calculated using total plant biomass, and the (**d**) mycorrhizal phosphorus responses (%) at each timepoint. Solid points and error bars represent the mean ± SE overlaid on top of the raw data points

Although abiotic factors would have affected the outcomes observed in this experiment, the homogenisation of the initial starting AM fungal community and the use of controlled environmental conditions would have significantly lessened their influence. At the very least, phylogenetic clustering under such conditions implies that the closely related fungal taxa may share particular traits that then confer membership and dominance of communities at the later stages of community assembly. It may further suggest that these traits are selected for by the plant host, and this selection drives the success of these taxa in the system. If this is the case, we might expect the host selection to confer a functional benefit. Our data support this hypothesis, as we found phylogenetic clustering corresponded with positive plant responses, reflecting functional advantages (Figs. 2, 3).

**Fig. 3:**
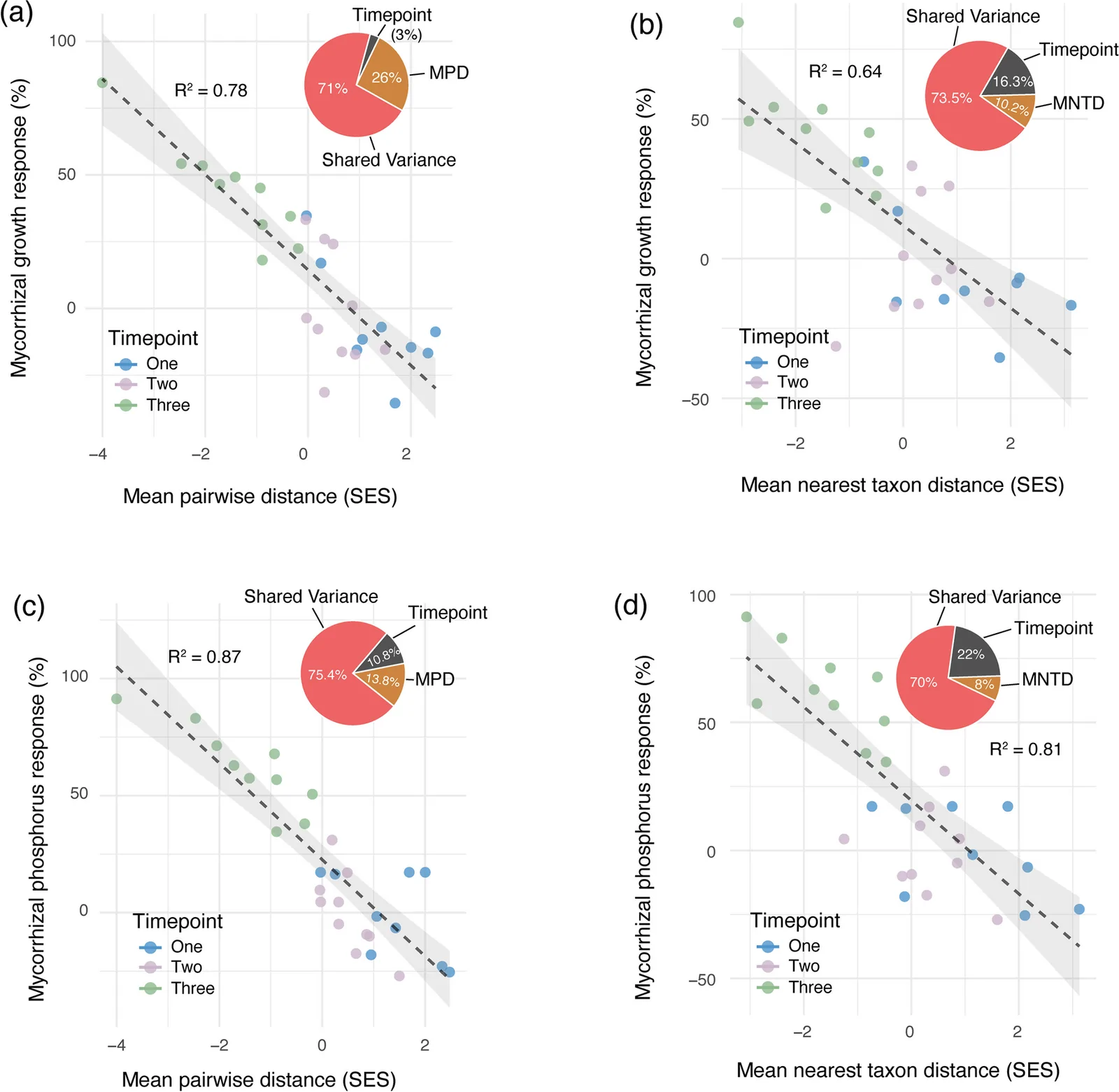
Relationships between phylogenetic diversity (showing the standardised effect sizes, SES) of root-colonising arbuscular mycorrhizal (AM) fungal communities and plant host responses to AM fungi. The relationships between mycorrhizal growth responses (%) and the (**a**) mean pairwise distances (MPD) and (**b**) mean nearest taxon distances (MNTD), and the relationships between the mycorrhizal phosphorus responses (%) and the (**c**) mean pairwise distance and the (**d**) mean nearest taxon distances. Each plot shows the amount of variation in the mycorrhizal growth responses (**a**, **b**) and mycorrhizal phosphorus responses (**c**, **d**) explained by the timepoint alone, the phylogenetic diversity (MPD or MNTD) alone, or shared by both timepoint and phylogenetic diversity. The coefficients of determination (R^2^) showing the total variation explained (including both timepoint and phylogenetic diversity as explanatory variables), are shown on each plot

The total plant biomass and phosphorus benefits conferred by AM fungi, as reflected in mycorrhizal growth responses (Fig. 2c) and mycorrhizal phosphorus responses (Fig. 2d), revealed that plants derived little growth or nutrient advantages from the AM fungi during the first and second timepoints. At the third timepoint, however, plants displayed significantly enhanced growth and phosphorus uptake in response to AM fungi (Fig. 2c, d). This coincided with the strong positive associations of Glomeraceae taxa with timepoint three (Fig. 1a) and the phylogenetic clustering of the fungal communities. Additionally, at this timepoint, a notable increase in the proportion of arbuscule structures within roots was also observed (Fig. S4d). Since arbuscules are the primary fungal structures involved in nutrient and carbon exchange between the host and fungi [2], a shift towards increased arbuscular colonisation may suggest an enhanced transfer of resources between the symbiotic partners. However, we acknowledge that arbuscule frequency, which can fluctuate significantly over time [2], can be a coarse measure for symbiotic function.

It is noteworthy that our results demonstrate a clear increase in the dominance of Glomeraceae taxa within communities over time (Fig. 1a). Glomeraceae are often characterised as putatively ruderal and disturbance-tolerant fungi that are fast-growing, and comparatively less nutritionally beneficial to hosts than other slower-growing fungal taxa [18, 36]. As ruderals, these AM fungi would be expected to colonise new habitats early; yet here we found their dominance later in community development. As such, it is less likely the strong succession patterns we observed are explained by phylogenetically related fungal traits that simply allow them to grow faster and access the root, but rather that their shared traits contribute to better host-fungal compatibility. That said, it remains possible that these fungi may simply possess certain traits, shared among phylogenetically similar taxa, that allow them to more easily colonise this root system. Still, the strong association between phylogenetic clustering and functional plant benefits suggests that host selection is a stronger influence of assembly through time than passive colonisation.

The ruderal characteristics of Glomeraceae taxa may indeed make them more suitable symbiotic partners for an agricultural crop selectively bred to have fast growth rates, particularly here in the context of a pot experiment which inherently represents a significant disturbance [37, 38]. It is also important to note that assigning life history strategies to particular AM fungal lineages still remains fraught with uncertainty [20] as comprehensive trait data across AM fungal taxa are still lacking. Although some studies suggest that certain AM fungal groups may exhibit distinct suites of traits [19, 39], confidently assigning lineages to a particular life history strategy is still premature.

We found strong relationships between the phylogenetic clustering of AM fungal communities and the growth and phosphorus benefits conferred by the symbiosis (Fig. 3). Both mean pairwise distances and mean nearest taxon distances exhibited significant and strong correlations with mycorrhizal growth and phosphorus responses. Variance partitioning revealed that the amount of variation in mycorrhizal growth responses explained by phylogenetic diversity alone reached as high as 26% (for mean pairwise distances), whereas time alone accounted for only 3% (Fig. 3a). While the amount of explained variation in mycorrhizal growth and phosphorus responses varied (Fig. 3a-d), the timepoint alone did not explain more than 22% of plant responses in any given instance. These results provide evidence that the temporal phylogenetic clustering of AM fungal communities within plant roots can be a key driver of the functional benefits the host derives from the symbiosis.

Our results indicate that selective processes in the roots can lead to positive outcomes for the host plant. However, we want to stress that strong host selection does not necessarily lead to positive outcomes. For example, evidence from plant-soil feedback experiments show that hosts can foster AM fungal communities that are beneficial [40, 41] but also communities that can negatively impact conspecific plants [42]. Thus, the nature of these interactions is highly context dependant, both on the effect of the AM fungal communities on the host [5], and the degree of host influence on AM fungal community assembly in roots. Since our study focuses on a single crop species, future research should assess the temporal assembly of root-colonising AM fungi across a wider range of host plants, not only across key agricultural species but also native plants. Understanding the capacity of crops to shape the assembly of beneficial AM fungal communities is essential for fully harnessing the functional benefits of this symbiosis.

## Supplementary Information

Below is the link to the electronic supplementary material.

ESM 1 (DOCX 580 KB)

## Data Availability

Data that support this study are openly available from the Figshare repository at the following doi: 10.6084/m9.figshare.26928256, which will become live upon article publication. Raw DNA sequencing data are available under NCBI BioProject accession number PRJNA1156093.
