# Supplementary material for: Increasing Phylogenetic Clustering of Arbuscular Mycorrhizal Fungal Communities in Roots Explains Enhanced Plant Growth and Phosphorus Uptake
Source: Microb Ecol. 2024 Nov 14;87:139. doi: 10.1007/s00248-024-02457-1 (PMC11564211; doi:10.1007/s00248-024-02457-1)
Supplement: Supplementary file 1 [file 248_2024_2457_MOESM1_ESM.docx]

Supplementary information for ‘*Increasing phylogenetic clustering of arbuscular mycorrhizal fungal communities in roots explains enhanced plant growth and phosphorus uptake’.*

**Supplementary Methods S1**

***Experimental set-up***

A factorial pot experiment was conducted with 60 individual plants of *Sorghum bicolor* L. Moench (cv. ‘MR Bazley’). The experiment involved two factors: AM fungi, with two levels (absence and presence of AM fungi), and timepoint, with three levels (timepoints one, two, and three). Prior to germinating seed directly in pots (3.7 L), seeds were surface sterilised using a 5% diluted commercial bleach solution (4% sodium hypochlorite).

The plants were cultivated in a homogenised mixture of sandy loam soil and washed river sand in a 30:70 ratio, which had been subjected to gamma irradiation at a dose of 50 kGy. For the AM fungal treatment, this soil-sand mixture was thoroughly blended with a soil inoculum at a 10% proportion. The inoculum consisted of field soils collected over a 12-month period from various agricultural and non-agricultural sites in Queensland and New South Wales, Australia. Following collection, these soils were air-dried, sieved, and stored with silica gel at temperatures ranging from 3°C to 5°C until needed. The soil inoculum was specifically prepared from a combination of soils known to support a high diversity of AM fungi at previous field samplings, to enhance the likelihood of a diverse range of root-colonising AM fungal taxa. For the treatment excluding AM fungi, the sterile soil-sand mix was combined with an equivalent 10% of autoclaved soil inoculum. Additionally, each pot received 200 mL of microbial liquid filtrate, which was obtained by washing the soil inoculum and filtering it through sieves with a pore size of 20 µm, ensuring consistency in the non-AM fungal microbial communities across all treatments. A 10 g initial application of low-phosphorus fertilizer, specifically Osmocote Native Controlled Release Fertilizer (The Scotts Company LLC), was uniformly mixed into all pots.

Plants were watered as needed, with soil moisture monitored every three days using a soil moisture meter to maintain consistent conditions across all pots. The greenhouse environment was maintained at day/night temperatures of 26°C and 19°C, respectively, with a 12-hour photoperiod. To mitigate any spatial effects, pots were randomly rearranged within the greenhouse every two weeks. Plants were harvested at timepoints one (4 weeks), two (8 weeks), and three (12 weeks). Timepoints all fell within vegetative growth stages of the crop, with the final timepoint (three), occurring before flowering. At each harvest, the roots and above-ground tissues were separated. A 1 g subsample of root tissue was taken and stored in 50% ethanol for subsequent assessment of mycorrhizal fungal colonisation, while the remaining plant tissues were dried at 38°C. Biomass was recorded, and all above-ground tissue was ground to a fine powder using a ball mill (mixer-mill MM400; Retsch, Haan, Germany) and homogenised prior to chemical analyses, while dried root tissue was retained for downstream DNA extractions and sequencing.

***Chemistry and colonisation of roots***

Phosphorus (P) concentrations in plants were assessed using inductively coupled plasma (ICP) spectroscopy following digestion with nitric acid [1]. Carbon concentrations were determined via the high-temperature combustion method using a LECO elemental analyser. For the assessment of mycorrhizal fungal colonisation, root samples stored in ethanol were cleared with 10% KOH at 90°C for 15 minutes, then stained with 5% ink-vinegar at 90°C for 20 minutes. The root fragments were mounted on glass slides with glycerin under a cover slip and examined microscopically using the intersect method at 200× magnification, with 100 intersections analysed per sample [2]. No colonisation of roots were detected in the roots samples taken from the ‘No AM fungi’ plants.

***Sequencing and bioinformatics***

DNA was extracted from 70 mg of dried root samples utilising the DNeasy Powersoil Pro Kit (Qiagen, GmbH). The sequencing process was carried out via the liquid handling pipeline at Western Sydney University’s Next-Generation Sequencing Facility. DNA purification was performed using Agencourt AMPure XP Beads (Beckman Coulter), and the quality of the purified DNA was subsequently assessed with Quant-iT™ PicoGreen fluorescence-based analysis (ThermoFisher Scientific). The purified DNA then underwent amplification using polymerase chain reaction (PCR) targeting the small-subunit (SSU) ribosomal RNA gene using AM fungal-specific primers [3] for the SSU ribosomal RNA gene using the WANDA (5′-CAGCCGCGGTAATTCCAGCT-3′; Dumbrell *et al.*, 2011) and AML2 (5′-GAACCCAAACACTTTGGTTTCC-3′; Lee *et al.*, 2008) primer set.

To process the sequencing data we used gDAT [6], a bioinformatic pipeline specifically optimised for analysis of SSU amplicons from AM fungi. We followed the defaults as outlined in Vasar et al.[6], but in short, the raw reads were retained if they carried the correct primer sequences (WANDA and AML2; allowing one mismatch for each), had an average quality of at least 30, and orphan reads were removed. Chimera checking was conducted with *vsearch* [7] in reference mode which checked each sequence individually against the Maarj*AM* database [8]. The AM fungal sequences, and their taxonomies, were identified to virtual taxa (VT) using BLAST+ [9] and the Maarj*AM* database as a reference with at least 99% identity and 95% alignment thresholds.

***Statistics***

All analyses were conducted using R version 4.3.3 (R Core Team, 2024).

To examine the AM fungal community assembly processes through time we used the joint species distribution Bayesian framework, Hierarchical Modelling of Species Communities (HMSC; [11]). Our fixed predictors were time/timepoints (categorical with three levels), as well as the log-transformed sequencing depth (continuous variable). To account for the study design, we also included the random effect of sample number. Model fitting was with the *Hmsc* function from the ‘Hmsc’ R package, assuming the default prior distributions [11]. We sampled the posterior distribution using four Markov Chain Monte Carlo (MCMC) chains, each producing 250 samples after a thinning of 1000 iterations. Each chain performed a total of 300,000 iterations, which includes a burn-in of 50,000 iterations per chain, culminating in 1,200,000 iterations across all chains. We examined the MCMC convergence by examining the potential scale reduction factors of model parameter (Fig. S1; [12]). We applied variance partitioning to calculate the proportion of explained variance in VT occurrences attributed by each of our model predictors.

We calculated the phylogenetic diversity and structure of AM fungal communities by the mean pairwise distance (MPD), measuring the mean phylogenetic distance between all VT pairs in a community, and the mean nearest taxon distance (MNTD) to measure the distance between each VT and its nearest relative, all employing the 'picante' package [13, 14]. We further calculated standardised effect sizes (SES) by way of the *ses.mpd* and *ses.mntd* functions from the package [14]. Higher values suggest phylogenetic overdispersion, whereas lower values imply phylogenetic clustering [15]. We employed linear models and the *Anova* function (type = ‘III’) from the ‘car’ package to determine if these metrics differed along the timepoints [16]. Rarefaction curves comparing the number of sequences to number of VT identified exhibited plateauing for each sample, suggesting an adequate or near-adequate sampling depth to capture AM fungal VT diversity. To explore potential differences in beta diversity of communities across time, dissimilarity was visualised by principal coordinate analysis (PCoA) based on Bray-Curtis dissimilarity of communities using the ‘Phyloseq’ package [17]. To test for the effects of plant host and the time of harvest on Bray-Curtis dissimilarities, we used permutational multivariate ANOVA (perMANOVA) using *adonis2* from the R package ‘vegan’ [18]. The variation in community composition (based on Bray-Curtis distances) was assessed based on the distance to group (timepoint) centroid using the *betadisper* function and *permutest* function in ‘vegan’[18].

We used the ‘iNEXT’ package [19] in R to obtain extrapolation curves for AM fungal VT richness and Shannon diversity (Hill numbers q = 0, 1). This was based on the number of sampling units with a 95% confidence interval set to 999 replications of bootstrap resampling. The differences in extrapolated VT richness and Shannon diversity among timepoints was examined by fitting linear models and running *Anova* from the ‘car’ package.

To explore the effects of the AM symbiosis on the host plants at the three time points, we calculated the mycorrhizal growth response (MGR) using the total plant biomass, and mycorrhizal phosphorus responses (MPR). These mycorrhizal plant responses were calculated as ([*plant response − mean plant responses with No AM fungi]/mean plant responses with no AM fungi) x 100*, where the plant response was either the total biomass or foliar phosphorus concentration. To assess if these differed among the timepoints we again fitted linear models and *Anova* from the ‘car’ package. The same models were used to look at differences in total and arbuscular colonisation, and total biomass and phosphorus concentrations responses except the models also included the AM fungi as a categorical predictor along with time. Vesicular colonisation which was analysed using permutational ANOVA from the R package ‘lmPerm’ [20].

We also explored the relative importance of time and phylogenetic diversity of the AM fungal communities in explaining plant growth and phosphorus responses to the AM symbiosis. To do this we constructed a full linear model, using *lm* function, that included both the phylogenetic/community relatedness metric (i.e., mean pairwise distance or mean nearest taxon distance) and time point as predictors of the plant responses. This model was used to calculate the total variance explained by the predictors combined, expressed as the coefficient of determination (R²). Next, we quantified the unique variance explained by each predictor by fitting separate linear models for each predictor individually, where phylogenetic relatedness was the sole predictor and where timepoint was the sole predictor. The unique contribution of each predictor to the variance was determined by subtracting the shared variance (i.e., the overlap in variance explained by both predictors) from the total variance explained by each individual predictor. The shared variance between community relatedness and time point was computed by subtracting the R² of the full model from the sum of the R² values of the individual models. This shared variance represents the portion of the total variance in the plant response (either growth or phosphorus responses) that is jointly explained by both phylogenetic/community relatedness and time.

**REFERENCES**

1.	Zarcinas BA, Cartwright B, Spouncer LR (1987) Nitric acid digestion and multi‐element analysis of plant material by inductively coupled plasma spectrometry. Communications in Soil Science and Plant Analysis 18:131–146. https://doi.org/10.1080/00103628709367806

2.	McGonigle TP, Miller MH, Evans DG, et al (1990) A new method which gives an objective measure of colonization of roots by vesicular—arbuscular mycorrhizal fungi. New Phytol 115:495–501. https://doi.org/10.1111/j.1469-8137.1990.tb00476.x

3.	Lekberg Y, Vasar M, Bullington LS, et al (2018) More bang for the buck? Can arbuscular mycorrhizal fungal communities be characterized adequately alongside other fungi using general fungal primers? New Phytologist 220:971–976. https://doi.org/10.1111/nph.15035

4.	Dumbrell AJ, Ashton PD, Aziz N, et al (2011) Distinct seasonal assemblages of arbuscular mycorrhizal fungi revealed by massively parallel pyrosequencing. New Phytologist 190:794–804. https://doi.org/10.1111/j.1469-8137.2010.03636.x

5.	Lee J, Lee S, Young JPW (2008) Improved PCR primers for the detection and identification of arbuscular mycorrhizal fungi. FEMS Microbiol Ecol 65:339–349. https://doi.org/10.1111/j.1574-6941.2008.00531.x

6.	Vasar M, Davison J, Neuenkamp L, et al (2021) User-friendly bioinformatics pipeline gDAT (graphical downstream analysis tool) for analysing rDNA sequences. Molecular Ecology Resources 21:1380–1392. https://doi.org/10.1111/1755-0998.13340

7.	Rognes T, Flouri T, Nichols B, et al (2016) VSEARCH: a versatile open source tool for metagenomics. PeerJ 4:e2584. https://doi.org/10.7717/peerj.2584

8.	Öpik M, Vanatoa A, Vanatoa E, et al (2010) The online database MaarjAM reveals global and ecosystemic distribution patterns in arbuscular mycorrhizal fungi (Glomeromycota). New Phytologist 188:223–241. https://doi.org/10.1111/j.1469-8137.2010.03334.x

9.	Camacho C, Coulouris G, Avagyan V, et al (2009) BLAST+: architecture and applications. BMC Bioinformatics 10:421. https://doi.org/10.1186/1471-2105-10-421

10.	R Core Team (2024) R: A Language and Environment for Statistical Computing. R Foundation for Statistical Computing, Vienna, Austria

11.	Ovaskainen O, Abrego N (2020) Joint Species Distribution Modelling: With Applications in R. Cambridge University Press

12.	Gelman A, Rubin DB (1992) Inference from iterative simulation using multiple sequences. Statistical Science 7:457–472. https://doi.org/10.1214/ss/1177011136

13.	Webb CO, Ackerly DD, McPeek MA, Donoghue MJ (2002) Phylogenies and community ecology. Annual Review of Ecology and Systematics 33:475–505

14.	Kembel SW, Cowan PD, Helmus MR, et al (2010) Picante: R tools for integrating phylogenies and ecology. Bioinformatics 26:1463–1464

15.	Pausas JG, Verdú M (2010) The jungle of methods for evaluating phenotypic and phylogenetic structure of communities. BioScience 60:614–625. https://doi.org/10.1525/bio.2010.60.8.7

16.	Fox J, Weisberg S (2011) An R Companion to Applied Regression, Second edition. Sage Publications, Thousand Oaks, CA

17.	McMurdie PJ, Holmes S (2013) phyloseq: An R Package for Reproducible Interactive Analysis and Graphics of Microbiome Census Data. PLOS ONE 8:e61217. https://doi.org/10.1371/journal.pone.0061217

18.	Oksanen J, Blanchet FG, Legendre P, et al (2015) vegan: Community Ecology Package. http://CRAN.R-project.org/package=vegan

19.	Hsieh TC, Ma KH, Chao A (2016) iNEXT: an R package for rarefaction and extrapolation of species diversity (Hill numbers). Methods in Ecology and Evolution 7:1451–1456. https://doi.org/10.1111/2041-210X.12613

20.	Wheeler B, Torchiano M (2016) lmPerm: Permutation Tests for Linear Models. R package version 2.1.0. https://CRAN.R-project.org/package=lmPerm

**Table S1.** Nutrient analysis of soil used in the pot experiment. Soil analysis performed prior to initiation of experiment. Analysis by CSBP Soil & Plant Analysis Laboratory, WA, Australia. Data shown are mean ± SE.

| Nutrient (units) |  |
| --- | --- |
| Ammonium nitrogen (mg/kg) | 23.7 ± 1.11 |
| Nitrate nitrogen (mg/kg) | 12.22 ± 2.59 |
| Phosphorus Colwell (mg/kg) | 9.5 ± 1.36 |
| Total carbon (%) | 1.61 ± 0.15 |
| Total nitrogen (%) | 0.12 ± 0.05 |
| pH | 5.84 ± 0.14 |

**Table S2**. Model results from general linear models and ANOVAs for the effects time and AM fungi, and their interactions on plant biomass, root : shoot ratios, and foliar phosphorus concentrations. Effect of timepoint on the mycorrhizal growth responses (MGR) and mycorrhizal phosphorus responses (MPR),total colonisation of plant roots, arbuscular and vesicular colonisation, the ratio of arbuscular colonisation to total colonisation, the mean pairwise distances (standardised effect sizes), mean nearest taxon distances (standardised effect sizes), extrapolated (Hill = 0) species richness, extrapolated (Hill = 1) Shannon, and community dispersion. All AM fungal response variables only relate to those plants with AM fungi. Significant impacts (*P* < 0.05) are indicated in bold.

|  | **Timepoint** | | **AM fungi** | | **Timepoint : AM fungi** | |
| --- | --- | --- | --- | --- | --- | --- |
|  | F _2,54_ | *P* | F _1,54_ | *P* | F _2,54_ | *P* |
|  |  |  |  |  |  |  |
| Total biomass | **435.6** | **<0.001** | **14.55** | **<0.001** | **31.51** | **<0.001** |
| Root : Shoot* | **27.08** | **<0.001** | **5.48** | **<0.05** | **4.13** | **<0.05** |
| Phosphorus concentration | **43.12** | **<0.001** | **20.77** | **<0.001** | **20.69** | **<0.001** |
|  | F _2,27_ | *P* |  |  |  |  |
|  |  |  |  |  |  |  |
| Mycorrhizal growth response | **15.05** | **<0.001** |  |  |  |  |
| Mycorrhizal phosphorus response | **40.06** | **<0.001** |  |  |  |  |
| Total colonisation | **71.1** | **<0.001** |  |  |  |  |
| Arbuscular colonisation | **114.2** | **<0.001** |  |  |  |  |
| Vesicular colonisation † | **2.66** | **<0.01** |  |  |  |  |
| Arbuscular / Total colonisation | **19.13** | **<0.001** |  |  |  |  |
| Mean pairwise distance (SES) | **26.86** | **<0.001** |  |  |  |  |
| Mean nearest taxon distance (SES) | **17.74** | **<0.001** |  |  |  |  |
| Extrapolated VT richness * | **3.59** | **<0.05** |  |  |  |  |
| Extrapolated Shannon diversity | 1.78 | 0.19 |  |  |  |  |
| Bray-Curtis dissimilarity ‡ | 1.09 | 0.27 |  |  |  |  |
| Dispersion (Bray-Curtis) † | 4.45 | **<0.05** |  |  |  |  |

* Log transformed

† permutational ANOVA

‡ PERMANOVA

**Figure S1**. The diagnostics for Markov Chain Monte Carlos (MCMC) convergence via potential scale reduction factors (psrf) for the HMSC occurrence model of arbuscular mycorrhizal (AM) fungal communities in roots. Histograms show the distribution of psrf values, with a focus on ensuring values are near 1.0, which implies reliable parameter estimation [12].

**Figure S2**. (**a**) Extrapolated arbuscular mycorrhizal (AM) fungal virtual taxon (VT) richness (Hill number q=0), (**b**) extrapolated Shannon diversity (Hill number q=1), and (**c**) principal coordinate analysis of communities based on Bray-Curtis dissimilarity at three different timepoints. For (a,b) solid points and error bars represent the mean ± SE overlaid on top of the raw data points, for (c) points represent individual samples/communities, ellipses show 95% confidence intervals.

**Figure S3**. (**a**) Total, (**b**) arbuscular, and (**c**) vesicular colonisation of roots by arbuscular mycorrhizal (AM) fungi at three different timepoints (one, two, and three). Solid points and error bars represent the mean ± SE overlaid on top of the raw data points.

**Figure S4**. Effects of arbuscular mycorrhizal (AM) fungi on the (**a**) total biomass, (**b**) foliar phosphorus concentration, and (**c**) root : shoot biomass ratios of Sorghum bicolor at three different timepoints. (**d**) Ratio of arbuscular colonisation to the total colonisation of roots by AM fungi at three different time points. Solid points and error bars represent the mean ± SE overlaid on top of the raw data points.

**Figure S5. (a)** Relative abundance (%) of arbuscular mycorrhizal fungal families within the initial soil inoculum used in the pot experiment. **(b**) Observed virtual taxa (VT) richness within roots of plants harvested at timepoints one, two, and three. Values are mean and standard error overlaid on top of raw data points.
